# Folate-Appended Hydroxypropyl-β-Cyclodextrin Induces Autophagic Cell Death in Acute Myeloid Leukemia Cells

**DOI:** 10.3390/ijms242316720

**Published:** 2023-11-24

**Authors:** Yasushi Kubota, Toshimi Hoshiko, Taishi Higashi, Keiichi Motoyama, Seiji Okada, Shinya Kimura

**Affiliations:** 1 Division of Hematology, Respiratory Medicine and Oncology, Department of Internal Medicine, Faculty of Medicine, Saga University, Saga 849-8501, Japan; 1367088@g.iuhw.ac.jp; 2 Department of Transfusion Medicine and Cell Therapy, Saitama Medical Center, Saitama Medical University, Kawagoe 350-8550, Japan; 3 Graduate School of Pharmaceutical Sciences, Kumamoto University, Kumamoto 862-0973, Japan; higashit@kumamoto-u.ac.jp (T.H.); motoyama@kumamoto-u.ac.jp (K.M.); 4 Division of Hematopoiesis, Joint Research Center for Human Retrovirus Infection, Kumamoto 860-0811, Japan; okadas@kumamoto-u.ac.jp

**Keywords:** 2-hydroxypropyl-β-cyclodextrin, folic acid, folate receptor, acute myeloid leukemia, autophagy, molecular targeting, Venetoclax, cholesterol, mitochondria, metabolism

## Abstract

Acute myeloid leukemia (AML) is a heterogenous myeloid neoplasm that remains challenging to treat. Because intensive conventional chemotherapy reduces survival rates in elderly patients, drugs with lower toxicity and fewer side effects are needed urgently. 2-Hydroxypropyl-β-cyclodextrin (HP-β-CyD) is used clinically as a pharmaceutical excipient for poorly water-soluble drugs. Previously, we showed that HP-β-CyD exerts antitumor activity by disrupting cholesterol homeostasis. Recently, we developed folate-conjugated HP-β-CyD (FA-HP-β-CyD) and demonstrated its potential as a new antitumor agent that induces not only apoptosis, but also autophagic cell death; however, we do not know whether FA-HP-β-CyD exerts these effects against AML. Here, we investigated the effects of FA-HP-β-CyD on folate receptor (FR)-expressing AML cells. We found that the cytotoxic activity of FA-HP-β-CyD against AML cells was stronger than that of HP-β-CyD. Also, FA-HP-CyD induced the formation of autophagosomes in AML cell lines. FA-HP-β-CyD increased the inhibitory effects of cytarabine and a BCL-2-selective inhibitor, Venetoclax, which are commonly used treat elderly AML patients. Notably, FA-HP-β-CyD suppressed the proliferation of AML cells in BALB/c nude *recombinase-activating gene-2* (*Rag-2*)/*Janus kinase 3* (*Jak3*) double-deficient mice with AML. These results suggest that FA-HP-β-CyD acts as a potent anticancer agent for AML chemotherapy by regulating autophagy.

## 1. Introduction

Acute myeloid leukemia (AML) is a heterogeneous disease driven by chromosomal abnormalities and genetic mutations [1]. Although 80% of AML patients achieve complete remission with conventional chemotherapy [2], the majority eventually relapse unless they undergo allogeneic hematopoietic cell transplantation. The median age at AML diagnosis is 68 years [1]. At this age, AML patients often have several comorbidities that preclude intensive chemotherapy or allogeneic stem cell transplantation [3]; therefore, they will receive less intensive chemotherapy regimens such as hypomethylating agents (HMA) and low-dose cytarabine (LDAC) [4]. Venetoclax, a selective BCL-2 inhibitor, shows high response rates and durable remission, with a favorable safety profile, when used in combination with HMA or LDAC in AML patients who are ineligible for intensive chemotherapy [4,5,6]. Currently, combined treatment with Venetoclax plus HMA or LDAC is the standard treatment strategy for elderly AML patients [7]. However, toxicities such as grade 3/4 neutropenia, which requires dose reduction, occur in a number of patients [8]. In the treatment of AML, chromosomal abnormalities and genetic mutations have also been shown to be useful in predicting and stratifying prognosis. The presence of mutations such as *TP53* and *ASXL1* is recognized as a particularly poor prognosis, while CBF-AML with chromosomal abnormalities such as t(8;21)(q22;q22) are classified as having a favorable prognosis [9,10,11]. Therefore, more effective and less toxic agents are desirable.

Cyclodextrins (CyDs) are cyclic oligosaccharides with hydrophilic outer surfaces and a hydrophobic inner environment; CyDs can encapsulate lipophilic compounds in their internal cavities, thereby rendering them soluble in aqueous solutions [12]. Therefore, CyDs are used widely by the pharmaceutical industry to improve drug solubility and bioavailability [13,14]. In addition, CyDs interact with cell components such as cholesterol and phospholipids, and methyl-β-cyclodextrin (M-β-CyD) is used in experiments as a lipid raft disrupting agent (the compound extracts cholesterol and sphingolipids from cell membrane) [15,16,17,18]. 2-Hydroxypropyl-β-CyD (HP-β-CyD), a pharmaceutical excipient for poorly water-soluble drugs, was used in clinical trials to remove accumulated intracellular lipids in patients with Niemann-Pick type C disease [19,20]. Given that cellular cholesterol is abundant in many malignancies, we hypothesized that HP-β-CyD, which can remove cholesterol, could act as a novel anticancer agent by directly modulating cholesterol homeostasis in cancer cells. Previously, we showed that HP-β-CyD exerts antitumor activity against BCR-ABL-positive cells, but its lack of tumor cell selectivity raised concerns about systemic cytotoxicity [21]. Therefore, we synthesized folate-appended HP-β-CyD (FA-HP-β-CyD) [22] to increase tumor cell selectivity (folate receptors (FRs) are highly expressed by tumor cells) [23,24], and showed that FA-HP-β-CyD was not toxic to FR-negative cells; however, it induced both apoptosis and autophagic cell death in chronic myeloid leukemia (CML) cells [22].

The FR family consists of four different members (α, β, γ, and δ) [25]. FRα is expressed in epithelial tissues, and FRβ is expressed in myeloid lineage cells. Furthermore, both FRα and FRβ have been shown to be strongly expressed in rapidly dividing cells, such as cancer cells [26,27,28]. FRβ is known to be expressed at high levels in blast cells of patients with AML [27,29], and, for example, anti-FRβ monoclonal antibody has shown promising results as a targeted agent against AML [25]. Therefore, FA-HP-β-CyD is also expected to exert a strong antitumor effect against AML. Here, we evaluated the efficacy of FA-HP-β-CyD as a treatment for AML.

## 2. Results

### 2.1. AML Cell Lines Express High Levels of the FR

**Figure 1:**
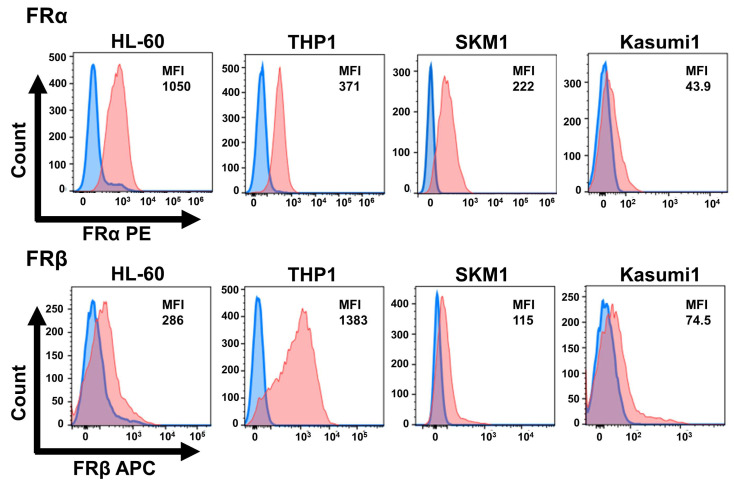
Cell surface expression of folate receptor (FR) α and β by AML cell lines. FRα expression by AML cells was measured by flow cytometry using an anti-FRα-PE antibody. Expression of FRβ was measured using an anti-FRβ-APC antibody. Blue: control; orange: sample stained with the anti-FRα or FRβ antibody. Median fluorescence intensity (MFI) for each cell lines is shown.

First, we performed flow cytometry analysis to investigate the expression of the FR in AML cell lines (Figure 1). FRα is strongly expressed in solid tumors, and FA-methyl-β-CyD has been shown to be effective against FRα-positive cells, including oral squamous carcinoma and melanoma [30,31]. Therefore, we examined the expression of FRα as well as FRβ in AML cells. HL-60 and SKM1 cells expressed FRα more strongly than FRβ, whereas THP1 and Kasumi 1 cells expressed FRβ more strongly than FRα. FRα was also expressed, but its expression was reported to be low in primary AML cells. This may be an artifact of the cell line, and further verification is needed. By contrast, FR expression by human bone marrow CD34-positive cells was very weak (Figure S1A,B).

### 2.2. FA-HP-β-CyD Inhibits Growth of AML Cell Lines More Potently Than HP-β-CyD

To examine the cytotoxic effect of FA-HP-β-CyD on AML cell lines, we used trypan blue assay. FA-HP-β-CyD (Figure S2) and HP-β-CyD (Figure S3) reduced the cell viability of the AML cell lines in a dose-dependent manner. Next, we evaluated the effects of FA-HP-β-CyD on the metabolic activity of AML cells. The 50% Inhibition Concentration (IC_50_) values of FA-HP-β-CyD were measured in AML cell lines. After 72 h of exposure to FA-HP-β-CyD, IC_50_ values ranged from 0.20 to 0.62 mM (Table 1). Detailed IC_50_ values including standard errors are shown in Table S1. The IC_50_ values of FA-HP-β-CyD were at least 8-fold lower than those of HP-β-CyD. Colony formation assays of normal human bone marrow CD34-positive progenitor cells confirmed that FA-HP-β-CyD had no effect on colony formation, but HP-β-CyD reduced colony numbers (Figure S1C).

### 2.3. FA-HP-β-CyD Inhibits Cell Growth by Inducing Apoptosis

**Figure 2:**
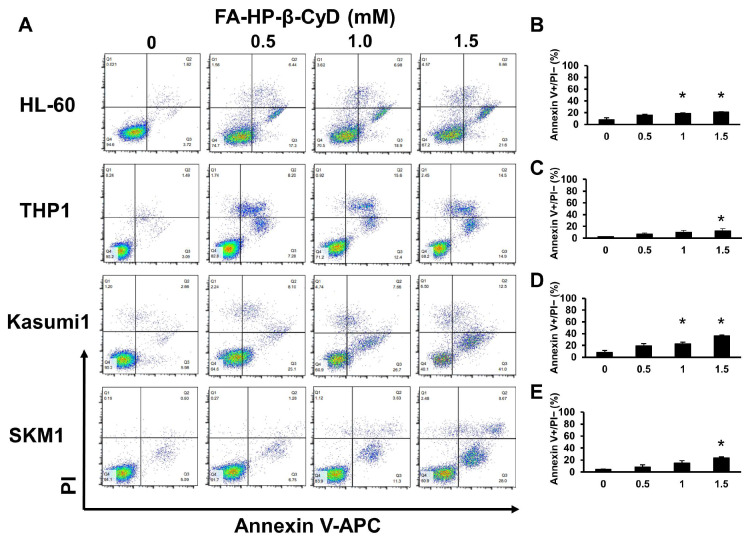
FA-HP-β-CyD induces apoptosis in HL-60, THP1, SKM1, and Kasumi1 cells. (**A**) HL-60, THP1, SKM1, and Kasumi1 cells were treated with 0 (medium only), 0.5, 1.0, and 1.5 mM of FA-HP-β-CyD for 72 h. After 72 h, cells were stained with Annexin V and PI. Representative FCM plots are shown (n = 3). (**B**–**E**) Percentage of Annexin V-positive PI-negative cells after exposure to FA-HP-β-CyD for 72 h. (**B**) HL-60, (**C**) THP1, (**D**) Kasumi1, (**E**) SKM1 cells. Data represent the mean ± SEM of three independent experiments. * *p* < 0.05.

Annexin V and propidium iodide (PI) staining were performed to evaluate apoptosis in FA-HP-β-CyD-treated AML cells. Exposure to FA-HP-β-CyD triggered early apoptosis in four AML cell lines in a dose-dependent manner (Figure 2). All AML cell lines exposed to HP-β-CyD underwent apoptosis (Figure S4). Cells exposed to 15 mM HP-β-CyD appeared to undergo necrosis.

### 2.4. FA-HP-β-CyD Does Not Affect the Cell Cycle Status of AML Cells

**Figure 3:**
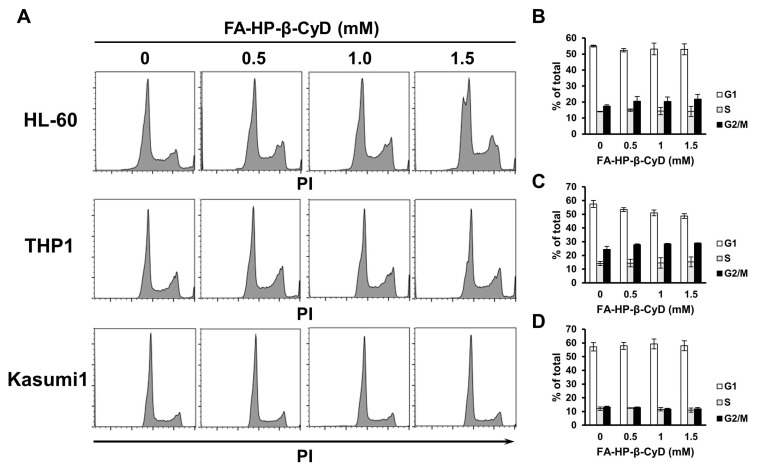
FA-HP-β-CyD does not affect cell cycle status in AML cells. HL-60, THP1 and Kasumi1 cells were treated for 24h with the indicated concentration of FA-HP-β-CyD and then subjected to flow cytometry analysis to detect PI-stained nuclei. (**A**) Representative flow cytometry histograms of PI-stained HL-60, THP1, and Kasumi1 cells. (**B**–**D**) Percentage of viable HL-60 (**B**), THP1 (**C**), and Kasumi1 cells (**D**) cells in G_0_/G_1_, S, or G_2_/M phase. Data represent the mean ± SEM of three independent experiments.

We then examined the effect of CyDs on the cell cycle. HL-60, THP1, and Kasumi1 cells exposed to HP-β-CyD showed a dose-dependent increase in the percentage of cells in G2/M phase (Figure S5). As the percentage of cells in G2/M increased, the percentage in the G1 phase decreased, whereas the percentage of cells in the S phase did not change significantly. However, FA-HP-β-CyD-treated AML cell lines showed no alteration in cell cycle status (Figure 3). This suggests that in addition to apoptosis, something other than cell cycle arrest underlies the potent antitumor effects of FA-HP-β-CyD. Furthermore, we also performed a cell cycle analysis of SKM1 before and after exposure to HP-β-CyD and found no change in cell cycle (Figure S6). Therefore, although there was no experimental data for SKM1, no change in the cell cycle status was predicted with FA-HP-β-CyD exposure.

### 2.5. FA-HP-β-CyD Induces Autophagosome Formation

**Figure 4:**
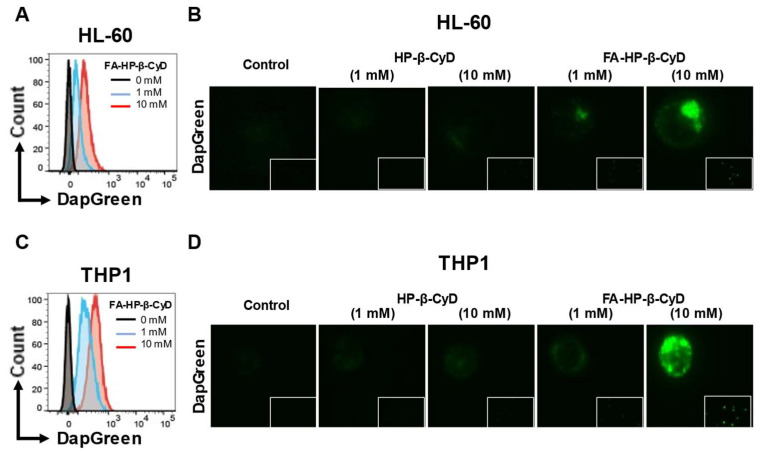
FA-HP-β-CyD induces autophagy in AML cells. (**A**,**B**) HL-60 cells were incubated at 37 °C for 30 min with 0.1 μM DAPGreen working solution and then treated with FA-HP-β-CyD (1 or 10 mM) for 2h. Then, fluorescence was observed by flow cytometry (**A**) or fluorescence microscopy (**B**, original magnification ×400). The blue and orange histograms in (**A**) show conditions treated with 1 mM FA-HP-β-CyD and 10 mM FA-HP-β-CyD, respectively. Gray histogram is a negative control. (**C**,**D**) THP1 cells were incubated at 37 °C for 30 min with 0.1 μM DAPGreen working solution and then treated with FA-HP-β-CyD (1 or 10 mM) for 2h. Then, fluorescence was observed by flow cytometry (**C**) or fluorescence microscopy (**D**, original magnification ×400). The blue and orange histograms in (**C**) show conditions treated with 1 mM FA-HP-β-CyD and 10 mM FA-HP-β-CyD, respectively. Gray histogram is a negative control.

The apoptosis-inducing effects of FA-HP-β-CyD do not completely explain its strong anti-proliferative effects of AML cells. Our previous studies suggest that the antitumor activity of FA-CyDs is mediated by autophagy [30,31,32]. Therefore, we investigated the effect of FA-HP-β-CyD on autophagosome formation in AML cells. For this purpose, we measured DAPGreen-derived fluorescence in response to FA-HP-β-CyD treatment. As shown by the fluorescence microscopy and flow cytometry results presented in Figure 4, the number of autophagic vacuoles in HL-60 and THP1 cells increased after treatment of FA-HP-β-CyD for 2 h.

### 2.6. Inhibiting Autophagy Dampens the Antitumor Activity of FA-HP-β-CyD

**Figure 5:**
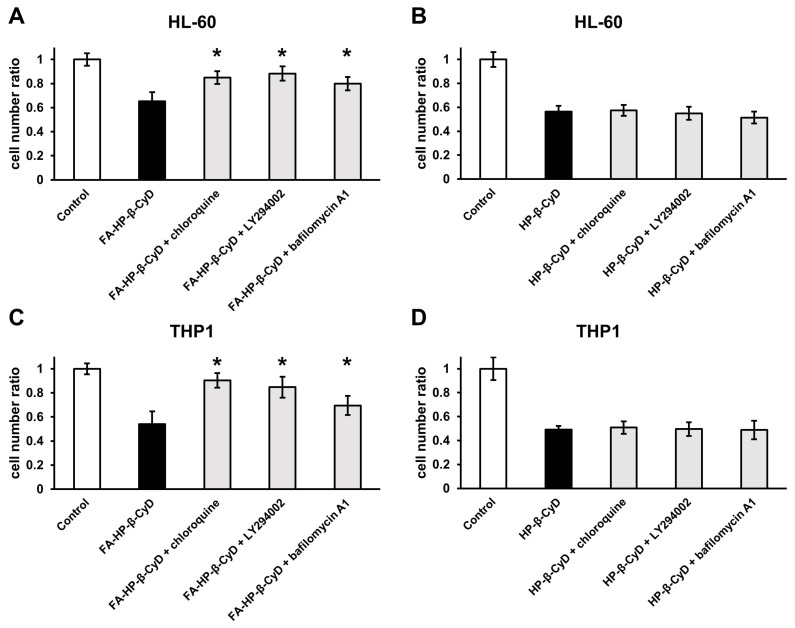
Autophagy inhibition in CyDs-treated AML cells. (**A**,**B**) Effects of chloroquine (20 μM), LY294002 (50 μM), and bafilomycin A1 (1 nM) on the antitumor activity of FA-HP-β-CyD (**A**) and HP-β-CyD (**B**) in HL-60 cells. (**C**,**D**) Effects of chloroquine (20 μM), LY294002 (50 μM), and bafilomycin A1 (1 nM) on the antitumor activity of FA-HP-β-CyD (**A**) and HP-β-CyD (**B**) in THP1 cells. Cells were treated with both 1 mM of FA-HP-β-CyD and 10 mM of HP-β-CyD at 37 °C for 2 h. Then, cells were incubated with the compounds for 24 h. Cell proliferation was assessed using Cell Counting Kit-8. Results are presented as the mean ± SEM (n = 3). * *p* < 0.05, compared with FA-HP-β-CyD without inhibitor.

Next, we examined the role of autophagy in the antitumor activity of FA-HP-β-CyD by treating AML cells with autophagy inhibitors bafilomycin A1, chloroquine, and LY294002. Bafilomycin A1 and chloroquine inhibit endosomal acidification, autophagosome-lysosome fusion, and lysosomal proteolysis, whereas LY294002 is a PI3K inhibitor. The viability of AML cells treated with FA-HP-β-CyD in the presence of autophagy inhibitors was higher than that in the presence of FA-HP-β-CyD alone (Figure 5A,C). By contrast, the survival of the cells treated with HP-β-CyD was not affected by the presence or absence of autophagy inhibitors (Figure 5B,D). These data indicate that FA-HP-β-CyD may cause AML cells to undergo autophagic cell death.

### 2.7. FA-HP-β-CyD Acts Synergistically to Enhance the Effects of Ara-C and Venetoclax

**Figure 6:**
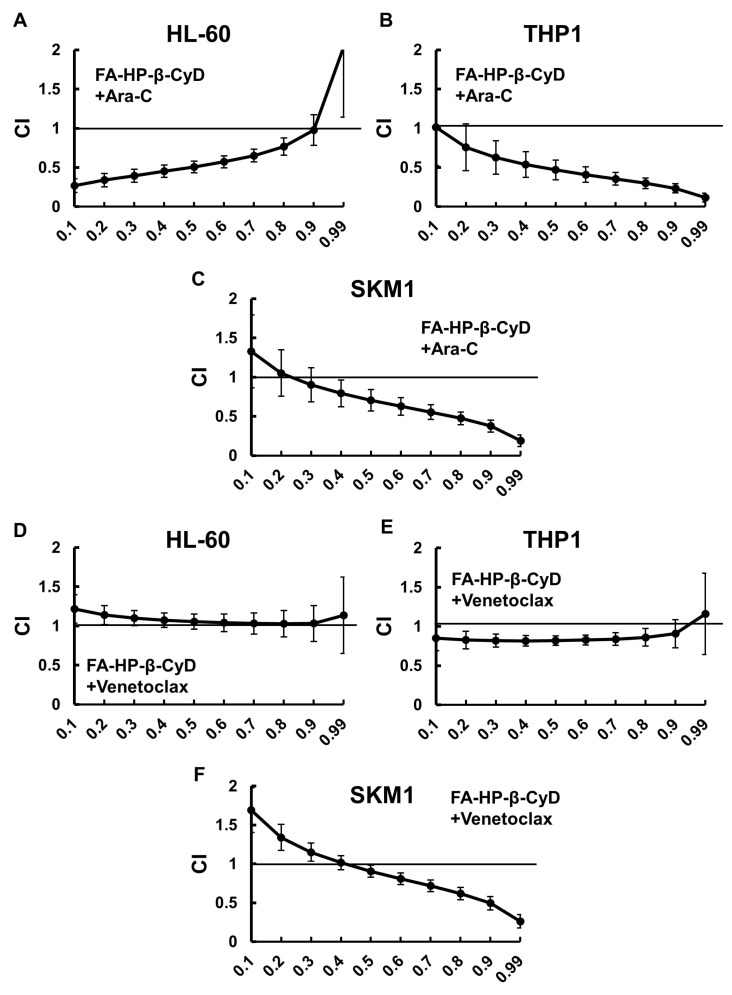
Combined effects of FA-HP-β-CyD and Ara-C or Venetoclax. (**A**–**C**) FA-HP-β-CyD was combined with Ara-C. (**D**–**F**) FA-HP-β-CyD was combined with Venetoclax. Cells were incubated for 72 h with five concentrations (0.25-, 0.5-, 0.75-, 1.0-, 1.5-fold the IC_50_) of each agent, or both in combination, using the constant ratio design, followed by modified MTT assay (Section 4.4). The IC_50_ values of Ara-C for HL-60, THP1 and SKM1 cells were 0.495 μM, 7.19 μM and 0.6 µM, respectively, and those of Venetoclax were 50.3 nM, 441.8 nM and 1.1 µM, respectively. The combination index (CI) was calculated using Calcusyn and plotted as a function of the fraction affected (Fa). For example, 50% growth inhibition would result in a Fa of 0.5. The synergistic (CI < 1), additive (CI = 1), or antagonistic (CI > 1) effects of combining multiple equal concentrations of drugs was assessed. Data are presented as the mean ± SEM of three independent experiments.

Next, we investigated the effect of combination treatment with FA-HP-β-CyD and Ara-C or Venetoclax on AML cell lines. To do this, we used a modified methyl-thiazolyl-diphenyl-tetrazolium (MTT) assay with five concentrations (0.25, 0.5, 0.75, 1.0, and 1.5-fold higher than the IC_50_) of each agent, or a combination of the two at a constant ratio. The IC_50_ values obtained from the experiments above were used (Figure S7). The IC_50_ values of Ara-C for HL-60, THP1 and SKM1 cells were 0.495 μM, 7.19 μM and 0.6 µM, respectively, and those of Venetoclax were 50.3 nM, 441.8 nM and 1.1 µM, respectively. The combination index (CI) and Fa values for each dilution were calculated using CalcuSyn software (version 1.1.1), as reported previously (Figures S8–S10) [22,33]. Dose–effect and CI-Fa plots illustrating the effect of the combinations of FA-HP-β-CyD and Ara-C or Venetoclax are presented in Figure 6. The combined treatment with FA-HP-β-CyD plus Ara-C or Venetoclax had a stronger growth inhibitory effect than either agent alone. Mathematical analyses of the data presented in the CI-Fa plots are shown (Figure 6). As shown in Table 2 and Table 3, the CI values at Fa 0.5 of the FA-HP-β-CyD with Ara-C or Venetoclax combinations were 0.507 and 1.056, respectively, in HL-60 cells; 0.469 and 0.820, respectively, in THP1 cells; and 0.706 and 0.905, respectively, in SKM1 cells. These observations indicate that FA-HP-β-CyD acts synergistically with Ara-C. The FA-HP-β-CyD/Venetoclax combination also showed synergistic effects in both THP1 and SKM1 cells. We also examined the effects of a combined treatment with HP-β-CyD plus Ara-C or Venetoclax. The combination of HP-β-CyD and Venetoclax acted antagonistically in all three AML cell lines examined, as indicated by CI values consistently being >1.0 ± 1 SD for all fractions, although Ara-C and HP-β-CyD showed additive effects in THP1 cells (Figure S11).

### 2.8. FA-HP-β-CyD Prolongs Survival of Acute Myeloid Leukemia Xenografts

**Figure 7:**
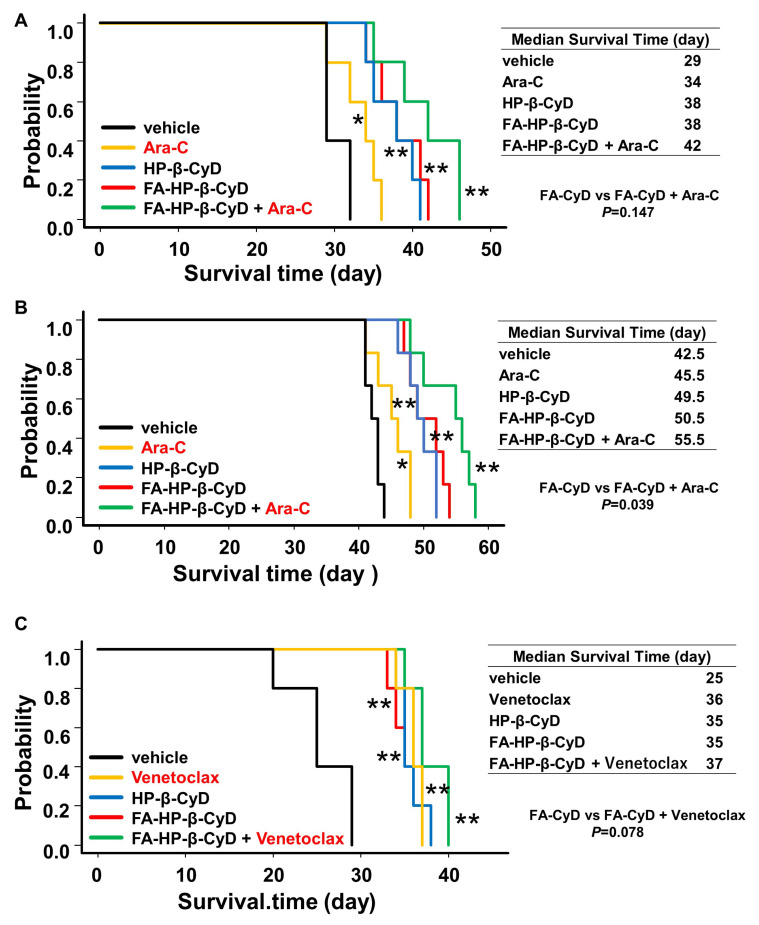
Effect of FA-HP-β-CyD on survival in leukemia mouse models and its effect in combination with Ara-C or Venetoclax. (**A**,**B**) Survival of mice transplanted with HL-60 cells (**A**) and THP12 cells (**B**). In these groups, the effect of Ara-C combination was also evaluated. HL-60 cells (5 × 10^6^) or THP1 cells (5 × 10^6^) were injected into BRJ mice. Three days later, mice received 200 μL of vehicle, 150 mM (2086.5 mg/kg) of HP-β-CyD, or 15 mM (249 mg/kg) of FA-HP-β-CyD via intraperitoneal injection (twice a day). Ara-C (100 mg/kg) was also injected intraperitoneally once a day. Administration continued for 20 days, and survival was checked daily. Black lines, yellow lines, blue lines, red lines, and green lines indicate survival of mice treated with vehicle, Ara-C, HP-β-CyD, FA-HP-β-CyD, and Ara-C plus FA-HP-β-CyD, respectively. (**C**) Survival curves of mice transplanted with HL-60 cells. In this group, the effect of Venetoclax combination was also evaluated. HL-60 cells (5×10^6^) were injected into BRJ mice. Three days after the injection, mice received 200 μL of vehicle, 150 mM (2086.5 mg/kg) of HP-β-CyD, or 15 mM (249 mg/kg) of FA-HP-β-CyD via intraperitoneal injection (twice a day). Venetoclax (25 mg/kg) was given daily by oral gavage. Administration was continued for 20 days, and survival was checked daily. Black lines, yellow lines, blue lines, red lines, and green lines indicate survival of mice treated with vehicle, Venetoclax, HP-β-CyD, FA-HP-β-CyD, and Venetoclax plus FA-HP-β-CyD, respectively. Survival data were analyzed using a log-rank nonparametric test and are shown as Kaplan–Meier survival curves (n = 6). * *p* < 0.05 and ** *p* <0.01 compared with vehicle group. Median survival time (day) for each group is shown.

The in vitro experiments showed that FA-HP-β-CyD markedly inhibited the growth of AML cells. To investigate the antitumor activity of FA-HP-β-CyD in vivo, we generated AML xenograft models and treated them with FA-HP-β-CyD, Ara-C, Venetoclax, or a combination of these. Two AML cell lines, HL-60 and THP1, were used as xenografts. The cells (5 × 10^6^) were transplanted into Balb/c Rag-2−/−Jak3−/− (BRJ) mice. Starting 3 days post-transplantation, mice were administered 200 μL of vehicle, 15 mM of FA-HP-β-CyD (249 mg/kg intraperitoneally (i.p.); b.i.d.), 150 mM of FA-HP-β-CyD (2086.5 mg/kg i.p.; b.i.d.), cytarabine (Ara-C, 100 mg/kg i.p.), or Venetoclax (25 mg/kg, oral gavage, once a day) for 20 days. Flow cytometry analysis was performed to detect human CD45+ cells in the bone marrow (BM) of control mice to confirm AML cell engraftment. HL-60-xenografted mice in the vehicle group died within 30 days, and THP1-xenografted died within 40 days. FA-HP-β-CyD-treated mice survived significantly longer than vehicle-injected mice (Figure 7). The combination of FA-HP-β-CyD and Ara-C prolonged survival of THP1-xenografted mice for significantly longer than FA-HP-CyD alone (Figure 7B, *p* = 0.039). Although not statistically significant, we noted a trend toward prolonged survival in HL-60 xenograft mice receiving the combination treatment (Figure 7A, *p* = 0.147). Survival of HL-60 xenograft mice receiving CyDs was similar to that of mice receiving Venetoclax (Figure 7C). Furthermore, there was no significant change in blood cell counts 8 days after intraperitoneal injection of both FA-HP-β-CyD and HP-β-CyD (compared with vehicle (PBS); the exception was the higher dose of HP-β-CyD (10,000 mg/kg b.i.d.)) (Table 4). These results suggest that FA-HP-β-CyD has potential as a novel drug candidate for the treatment of AML with negligible hemotoxicity.

## 3. Discussion

Here, we show that FA-HP-β-CyD inhibits AML cell proliferation more potently than HP-β-CyD. The mechanism by which FA-HP-β-CyD triggers death in AML cells may involve autophagy, as we showed previously for BCR-ABL-positive cells [22]. The combination of FA-HP-β-CyD plus Ara-C or Venetoclax had a synergistic or additive inhibitory effect on AML cells. In a mouse model of AML, FA-HP-β-CyD had a stronger inhibitory effect on AML progression than HP-β-CyD.

Our previous study showed that HP-β-CyD induced cell cycle arrest and apoptosis in tumor cells by disrupting cholesterol homeostasis. In the present study, HP-β-CyD but not FA HP-β-CyD induced cell cycle arrest in AML cells. Furthermore, the intraperitoneal injection of HP-β-CyD significantly prolonged the survival of BCR-ABL-induced leukemia mouse models [21]. However, since the tumor cell-targeting ability of HP-β-CyD is low, a strategy to increase tumor selectivity was considered. We focused on the fact that FRs are expressed by tumor cells at much higher levels than by normal cells, and so we synthesized FA-HP-β-CyD. In this study, we found that FA-HP-β-CyD not only induced apoptosis, but also autophagy-mediated cell death in AML cells. In our previous studies using FA-HP-β-CyD and FA-methyl-β-cyclodextrin [22,32], FA-CyDs entered cells and induced mitophagy, leading to autophagic cell death. Thus, we assume that the same phenomenon occurs in AML cells. In the present study, FA-HP-β-CyD was found to induce apoptosis in AML cells. This result was similar to the findings for CML cells [22]. The results of in vitro combination experiments suggest that FA-HP-β-CyD does not appear to be antagonistic to venetoclax, which induces apoptosis by inhibiting BCL-2. However, the mechanism of apoptosis induction by FA-HP-β-CyD remains unclear and will be the subject of future studies. Although the AML cell lines examined in the present study had the TP53 mutation, each lineage was different and differed greatly in the intensity of FR expression. Therefore, verification using a larger number of cell lines and primary AML cells is needed.

The role of autophagy in cancer is still controversial. Since autophagy is a mechanism that regulates cell death under stressful conditions, it is generally thought to protect leukemia cells during exposure to anticancer drugs; indeed, autophagy has been shown experimentally to be involved in resistance to various anticancer drugs. In addition, analyses of human AML and CML cells show that treatment with daunorubicin (DNR) or Ara-C activates autophagy, and that inhibiting autophagy increases the cytotoxic effects of DNR and Ara-C in vitro [34,35]. Furthermore, an in vivo model in which OCI-AML3 cells were transplanted into immunocompromised mice showed that the knockdown of ATG7, which is essential for autophagy, works synergistically with Ara-C, thereby confirming the involvement of autophagy in Ara-C resistance [36]. In contrast to its cytoprotective role, autophagy is also required for the antileukemic effects of certain drugs. Autophagy of acute promyelocytic leukemia cells is induced by exposure to retinoic acid or arsenic trioxide (ATO), and it is essential for the degradation of PML-RARα fusion protein by retinoic acid or ATO [37,38,39]. Similarly, autophagy is essential for degradation of BCR-ABL fusion protein by ATO in CML, and the inhibitory effects of ATO on colony-forming ability is reduced by lysosome inhibitors [40].

Autophagy is also implicated in the effects of histone deacetylase (HDAC) inhibitors. An analysis of Down syndrome-associated myeloid leukemia cell lines showed that HDAC inhibitors suppress autophagy by increasing the acetylation of autophagy-related proteins such as ATG7 and exhibit autotoxic activity against cells with low autophagic activity [41]. By contrast, HDAC inhibitors activate autophagy in cell lines carrying the AML1-ETO fusion gene; also, the effects of HDAC inhibitors on autophagy activity appear to vary according to cell type because the combination of HDAC inhibitors and chloroquine increases drug efficacy [42]. A recent study shows that decitabine activates autophagy in AML cells by down-regulating TP53-induced glycolysis and the apoptosis regulator TIGAR [43]. HDAC inhibitors are also important AML therapeutic drugs, and HDAC resistance is an urgent issue. However, it could not be fully investigated in the present study. Some groups demonstrated the induction of autophagic cell death in tumor cells, including colon, prostate, and breast, in response to various anticancer agents [44,45,46]. These data suggest that autophagy does not always play the same role during leukemia treatment. Thus, autophagy is a double-edged sword. Nevertheless, in the present study, FA-HP-β-CyD induced autophagic cell death in AML cells, as we previously showed in CML cells. Thus, FA-HP-β-CyD may be useful as an autophagy inducer for the treatment of leukemia. FA-HP-β-CyD may promote the activation of autophagy beyond leukemic cell homeostasis, resulting in cell death.

In AML cells, FA-HP-β-CyD showed a synergistic or additive growth inhibitory effect when used alongside Ara-C or Venetoclax. However, HP-β-CyD showed only antagonistic effects when combined with Ara-C or Venetoclax. One possible reason for the antagonistic effect of HP-β-CyD is that it may encapsulate Ara-C or Venetoclax, thereby reducing their efficacy. Indeed, attempts to form inclusion complexes between CyDs and drugs such as dasatinib and thymoquinone have been reported, but it is not clear whether the antileukemic effect is enhanced [47,48,49]. Another possibility is that HP-β-CyD disrupts the signaling platform for the drugs by removing cholesterol from lipid rafts in the cell membrane. Although the exact underlying mechanism remains unknown and needs to be verified, we speculate that FA-HP-β-CyD is incorporated into AML cells and induces autophagy without excessively disrupting lipid rafts, as we showed previously in BCR-ABL-positive cells [22].

A typical side effect of antileukemic drugs is cytopenia; however, administration of FA-HP-β-CyD had no adverse effects on white blood cell counts, hemoglobin concentrations, or platelet counts. Although long-term data are required, our current data suggest that FA-HP-β-CyD has the potential for use in elderly patients who are prone to complications, including infections caused by cytopenia.

Our study had limitations. We have not been able to assay with more cell lines. In particular, only two in vivo mouse models were possible. One reason for this is that it is still difficult to rapidly synthesize sufficient amounts of FA-HP-β-CyD. In the future, we would like to conduct experiments with more AML cell lines to demonstrate the robust efficacy of FA-HP-β-CyD on AML.

## 4. Materials and Methods

### 4.1. Reagents

HP-β-CyD was purchased from Tokyo Chemical Industry (Tokyo, Japan). The average molecular weight of HP-β-CyD was 1391. For in vitro use, the compound was dissolved in saline (Otsuka Pharmaceuticals, Tokyo, Japan) to a concentration of 500 mM. For in vivo use, HP-β-CyD was dissolved in saline to a concentration of 150 mM. The solutions were stored at 4 °C until use. FA-HP-β-CyD was prepared as previously described [22]. In brief, FA was conjugated to ethylenediamine (EDA) to introduce an amino group. FA-FDA was obtained by washing three times with acetonitrile, followed by suction filtration. Next, HP-β-CyD was activated with 1,1′-carbonyldiimidazole (CDI) to synthesize CDI-activated HP-β-CyD. Next, FA-EDA was reacted with CDI-activated HP-β-CyD to obtain FA-HP-β-CyD. The preparation was confirmed by MALDI-TOF-MS and 1H-NMR. Venetoclax was purchased from MedChemExpress (Monmouth Junction, NJ, USA) and formulated in phosal 50 mixture solution comprising 60% phosal 50 propylene glycol (H. Holstein), 30% polyethylene glycol 400, and 10% ethanol, as described previously [50].

### 4.2. Cell Lines and Cultures

SKM1 cells (established from the blast cells of a MDS patient) were purchased from the Japanese Collection of Research BioResources Cell Bank (Ibaraki, Osaka, Japan). AML cell lines, including HL-60, THP-1, and Kasumi-1, were purchased from the American Type Culture Collection (ATCC, Manassas, VA, USA).

Human bone marrow CD34 positive cells were purchased from LONZA (Bazel, Switzerland). FA-HP-β-CyD toxicity in normal CD34 positive cells was investigated using a standard methylcellulose culture assay. A total of 2 × 10^3^ cells were exposed to 0, 10 mM HP-β-CyD and 1 mM FA-HP-β-CyD in 1 mL MethoCult H4435 (Stem Cell Technologies, Vancouver, BC, Canada). After 1 week of culture, the number of colonies was counted using an inverted microscope. Data represent the mean number of colonies ± SEM (n = 3).

### 4.3. Mice

BALB/c *Rag-2*/*JAK3* double-deficient (BRJ) mice [51] were housed in a specific pathogen-free barrier facility and used for the xenograft transplantation experiments. All animal experiments were approved by the Institutional Review Board of Saga University and conducted in accordance with the institutional guidelines of animal care of Saga University.

### 4.4. Analysis of Cell Growth and Viability

The trypan blue dye exclusion method was used to evaluate cell viability. Cells (HL-60, THP1, SKM1 and Kasumi1 cells) were seeded in flat-bottomed 96-well plates at a density of 1 × 10^4^ cells in 100 µL medium per well and incubated with FA-HP-β-CyD (0, 0,1, 0.25, 0.5, 0.75, 1 mM) or HP-β-CyD (0, 1, 2.5, 5, 10 mM) for 72 h. Then, viable cells were counted. Cell proliferation was evaluated by a modified MTT assay using Cell Counting Kit-8 (Dojindo Molecular Technologies, Kumamoto, Japan), as previously described [21,22]. AML cells were cultured in 96-well plates at a density of 1 × 10^4^ cells in 100 µL medium per well and incubated with FA-HP-β-CyD or HP-β-CyD for 72 h. The half-maximal inhibitory concentration values (IC_50_) were determined by non-linear regression using CalcuSyn software version 1.1.1 (Biosoft, Cambridge, UK), as previously described [52]. After obtaining IC_50_ values for each agent, the antileukemic activity of FA-HP-β-CyD plus cytosine arabinoside (Ara-C) or Venetoclax was evaluated, as previously described [25]. Five concentrations (0.25-, 0.5-, 0.75-, 1.0-, and 1.5-fold the IC_50_) of FA-HP-β-CyD plus Ara-C or Venetoclax were used to treat AML cells. CalcuSyn was used to calculate the fraction affected (Fa, in which a value of 0.25 indicates 25% viable cells) and the CI. This method is capable of quantifying synergistic (CI < 1) and antagonistic (CI > 1) effects at different dose and effect levels.

### 4.5. Flow Cytometry Analysis

To detect expression of FR, suspensions of 2 × 10^6^ leukemia cells (HP-60, THP1, SKM1, and Kasumi1 cells) were incubated with a PE-conjugated anti-FOLR1 antibody, an APC-conjugated anti-human FR-antibody, and an APC-conjugated anti-mouse FR-antibody (all from BioLegend, San Diego, CA, USA). PI and Annexin V (BD Biosciences, San Jose, CA, USA) staining was performed to assess apoptosis, as previously described [53]. Cells were filtered through nylon mesh and assessed using a FACSVerse flow cytometer (BD Biosciences). Data were analyzed using FlowJo software 10.8.2 (Tree Star, Inc., Ashland, OR, USA).

### 4.6. Cell Cycle Analysis

Cell cycle analyses of AML cell lines (HL-60, THP1, SKM1, and Kasumi1 cells) were performed as described previously [21]. In brief, 1 × 10^6^ cells were treated with the indicated concentrations of CyDs. After 24 h of CyD treatment, cells were collected and fixed with 70% ethanol. Cells were then incubated at room temperature for 30 min with 0.1% Triton X-100/0.5% RNase A and stained with 50 μg/mL PI (BD Biosciences, San Jose, CA, USA). Cellular DNA content was analyzed by flow cytometry, and cell cycle profiles were determined in a FACS CantoII flow cytometer with CellQuest software (version 1.0.6, BD Biosciences). Data are presented as the mean ± SEM of three independent experiments.

### 4.7. Autophagy Detection

Autophagosome formation was evaluated using the DAPGreen-Autophagy Detection Kit (Dojindo Molecular Technologies). HL-60 and THP1 cells were cultured overnight in 6-well plates at a density of 5 × 10^5^ or 1 × 10^6^ cells. The cells were then washed once with culture medium and incubated with DAPGreen working solution at 37 °C for 30 min. After the cells were washed twice, CyDs (1 mM, 10 mM) were added to the wells and incubated for 2 h. The samples were analyzed using a flow cytometer (FACS Canto II) or a fluorescence microscope (Carl Zeiss, Jena, Germany).

### 4.8. Culture of Cells with Autophagy Inhibitors

HL-60 and THP1 cells (2 × 10^5^ cells in 100 µL of RPMI-1640 medium per well) were seeded in flat-bottomed 96-well plates (Greiner Labortechnik, Hamburg, Germany) and treated with both 1 mM of FA-HP-β-CyD and 10 mM of HP-β-CyD at 37 °C for 2 h. The cells were then pretreated for 24 h with the autophagy inhibitors chloroquine (20 µM), LY294002 (50 μM; Fujifilm Wako Pure Chemicals Co., Ltd., Osaka, Japan), or bafilomycin A1 (1 nM; Funakoshi, Tokyo, Japan). Cell proliferation was assessed using Cell Counting Kit-8. Data are presented as the mean ± SEM of three independent experiments.

### 4.9. Murine Leukemia Model

HL-60 cells or THP-1 cells were injected intravenously into BRJ mice. After 72 h, mice were injected intraperitoneally with 200 μL of vehicle, FA-HP-β-CyD (15 mM, 249 mg/kg), or HP-β-CyD (150 mM, 2086.5 mg/kg) for 20 consecutive days, and survival was monitored daily. Ara-C (100 mg/kg) was injected intraperitoneally for 5 consecutive days from 72 h after transplantation. Venetoclax (25 mg/kg) was orally administered for 20 consecutive days from 72 h after transplantation. Chimerism of human leukemic cells in the BM was determined by flow cytometry after double staining with FITC-conjugated anti-human CD45 (BD Bioscience) and PE/Cy7-conjugated anti-mouse CD45 (BioLegend) antibodies. All surgery was performed under sodium pentobarbital anesthesia to minimize pain. Mice were euthanized with ether when they became moribund or were unable to obtain food or water, as recommended by Saga University institutional guidelines. Survival data were analyzed using a log-rank nonparametric test and presented as Kaplan–Meier survival curves (n = 6 for each group).

### 4.10. Blood Counts

BRJ mice were injected intraperitoneally with vehicle, HP-β-CyD, or FA-HP-β-CyD every day for 7 days. At 24 h after the first injection, and at 8 days after the last of seven repeat injections, peripheral blood was collected from the tail vein and analyzed by an automated blood cell counter (Celltacα MEK-6500; NIHON KOHDEN, Tokyo, Japan). Data are presented as the mean ± SEM of three independent experiments.

### 4.11. Statistical Analysis

Data are presented as the mean ± SEM. An unpaired two-tailed Student’s *t*-test was used to analyze the data; *p* values < 0.05 were considered significant. To evaluate in vivo efficacy, survival curves were generated using the Kaplan–Meier method and compared using the log-rank test. All statistical analyses were performed using the EZR software package (version 1.61) [54].

## 5. Conclusions

In summary, we evaluated the potential of FA-HP-β-CyD as a novel treatment for AML treatment both in vitro and in vivo. FA-HP-β-CyD displayed greater anti-AML activity than HP-β-CyD in vitro. The antitumor activity of FA-HP-β-CyD may be mediated via both autophagy and apoptosis. FA-HP-β-CyD prolonged the survival of AML mouse models significantly when compared with HP-β-CyD and Ara-C. Further research is required to clarify the molecular mechanism underlying the antitumor activity of FA-HP-β-CyD and to evaluate its effect on AML stem cells. In the field of cancer chemotherapy, the acquisition of drug resistance is a challenge. FA-HP-β-CyD, which induces cell death through a non-apoptotic pathway, is a new therapeutic option.

## Figures and Tables

**Table 1:** IC_50_ values of FA-HP-β-CyD and HP-β-CyD in various AML cell lines.

	IC_50_ (mM)	
Cell Line	HP-β-CyD	FA-HP-β-CyD
HL-60	7.66	0.62
THP-1	9.05	0.32
SKM-1	9.44	0.2
Kasumi-1	3.14	0.3

Values represent the mean at least three independent experiments.

**Table 2:** Values of the Fa 0.5 from combined treatment with Ara-C.

	HP-β-CyD + Ara-C	FA-HP-β-CyD + Ara-C
HL-60	1.412	0.507
THP1	1.085	0.469
SKM1	1.299	0.706

Data are presented as the mean ± SEM of three independent experiments. Fa, fraction affected; Ara-C, cytarabine.

**Table 3:** Values of the Fa 0.5 from combined treatment with Venetoclax.

	HP-β-CyD + Venetoclax	FA-HP-β-CyD + Venetoclax
HL-60	2.013	1.056
THP1	1.415	0.82
SKM1	1.26	0.905

Data are presented as the mean ± SEM of three independent experiments. Fa, fraction affected.

**Table 4:** Peripheral blood cell count after intraperitoneal administration of CyDs to BRJ mice.

		HP-β-CyD (mg/kg)			FA-HP-β-CyD (mg/kg)		
	vehicle	2000	6000	10,000	200	600	1000
WBC (×10^2^/μL)	57.0 ± 10.67	44.7 ± 12.65	50.0 ± 2.34	30.5 ± 1.65 *	50.7 ± 13.36	52.5 ± 3.84	55.7 ± 14.28
Hb (g/dL)	15.1 ± 0.60	12.4 ± 0.67	14.3 ± 0.25	14.5 ± 0.78	13.8 ± 1.12	14.5 ± 0.27	14.7 ± 0.28
Plt (×10^4^/μL)	59.7 ± 11.0	64.5 ± 2.29	60.2 ± 10.69	75.5 ± 17.83	55.8 ± 15.67	61.1 ± 12.50	55.7 ± 18.21

Peripheral bood was collected from tail vein on Day 8 after the last of seven repeat injections of CyDs. Values are presented as the mean ± SEM (n = 3–4). * *p* < 0.05.

## Data Availability

The data presented in this study are available in the present article (and Supplementary Materials).

## Supplementary Materials

The following supporting information can be downloaded at: https://www.mdpi.com/article/10.3390/ijms242316720/s1.
