# Supplementary material for: Folate-Appended Hydroxypropyl-β-Cyclodextrin Induces Autophagic Cell Death in Acute Myeloid Leukemia Cells
Source: Int J Mol Sci. 2023 Nov 24;24(23):16720. doi: 10.3390/ijms242316720 (PMC10706821; doi:10.3390/ijms242316720)
Supplement: Supplementary file 1 [file ijms-24-16720-s001.zip › Table S1.pdf]

Table S1. IC_{50} values of FA-HP-β-CyD and HP-β-CyD in various AML cell lines (with SEM)

	IC_{50} (mM)	
**Cell Line**	**HP-β-CyD**	**FA-HP-β-CyD**
HL-60	7.66 ± 1.59	0.62 ± 0.07
THP-1	9.05 ± 0.27	0.32 ± 0.07
SKM-1	9.44 ± 0.66	0.20 ± 0.05
Kasumi-1	3.14 ± 0.42	0.30 ± 0.02

Values represent the mean ± SEM of at least three independent experiments.
