# Supplementary figures and images for: Folate-Appended Hydroxypropyl-β-Cyclodextrin Induces Autophagic Cell Death in Acute Myeloid Leukemia Cells
Source: Int J Mol Sci. 2023 Nov 24;24(23):16720. doi: 10.3390/ijms242316720 (PMC10706821; doi:10.3390/ijms242316720)

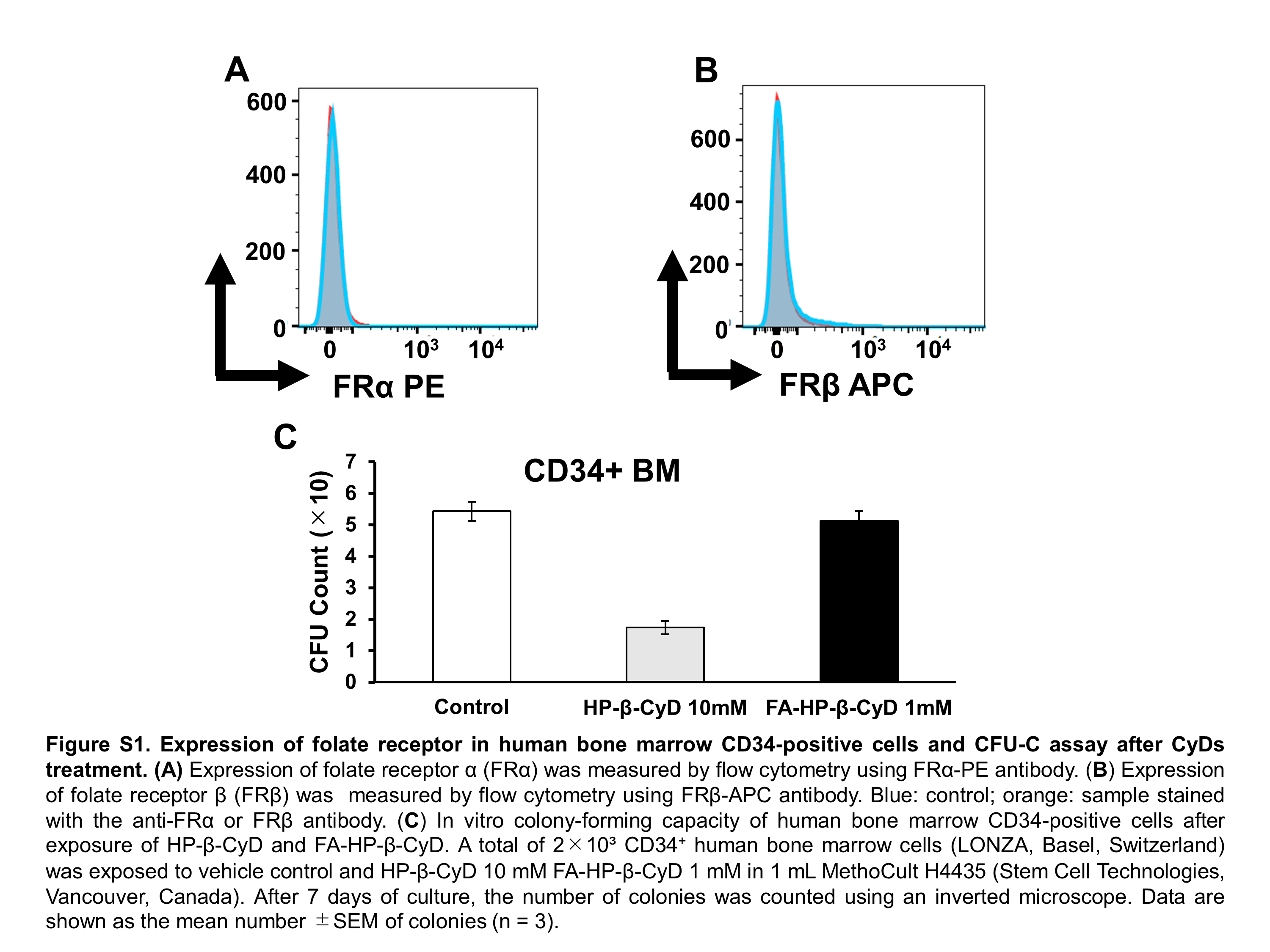

Supplement: Supplementary file 1 [file ijms-24-16720-s001.zip › Figure S1 proof.tif]

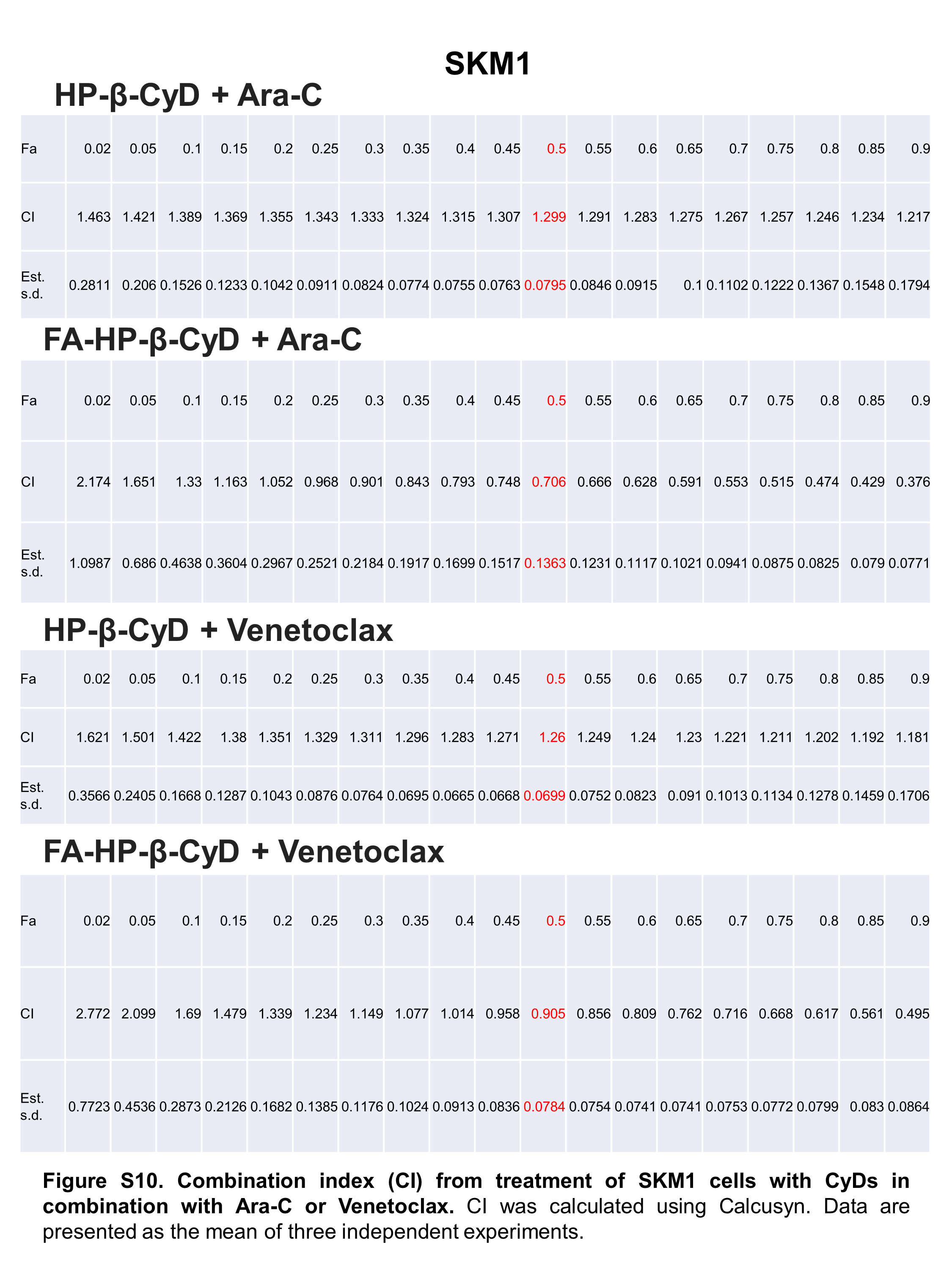

Supplement: Supplementary file 1 [file ijms-24-16720-s001.zip › Figure S10 proof.tif]

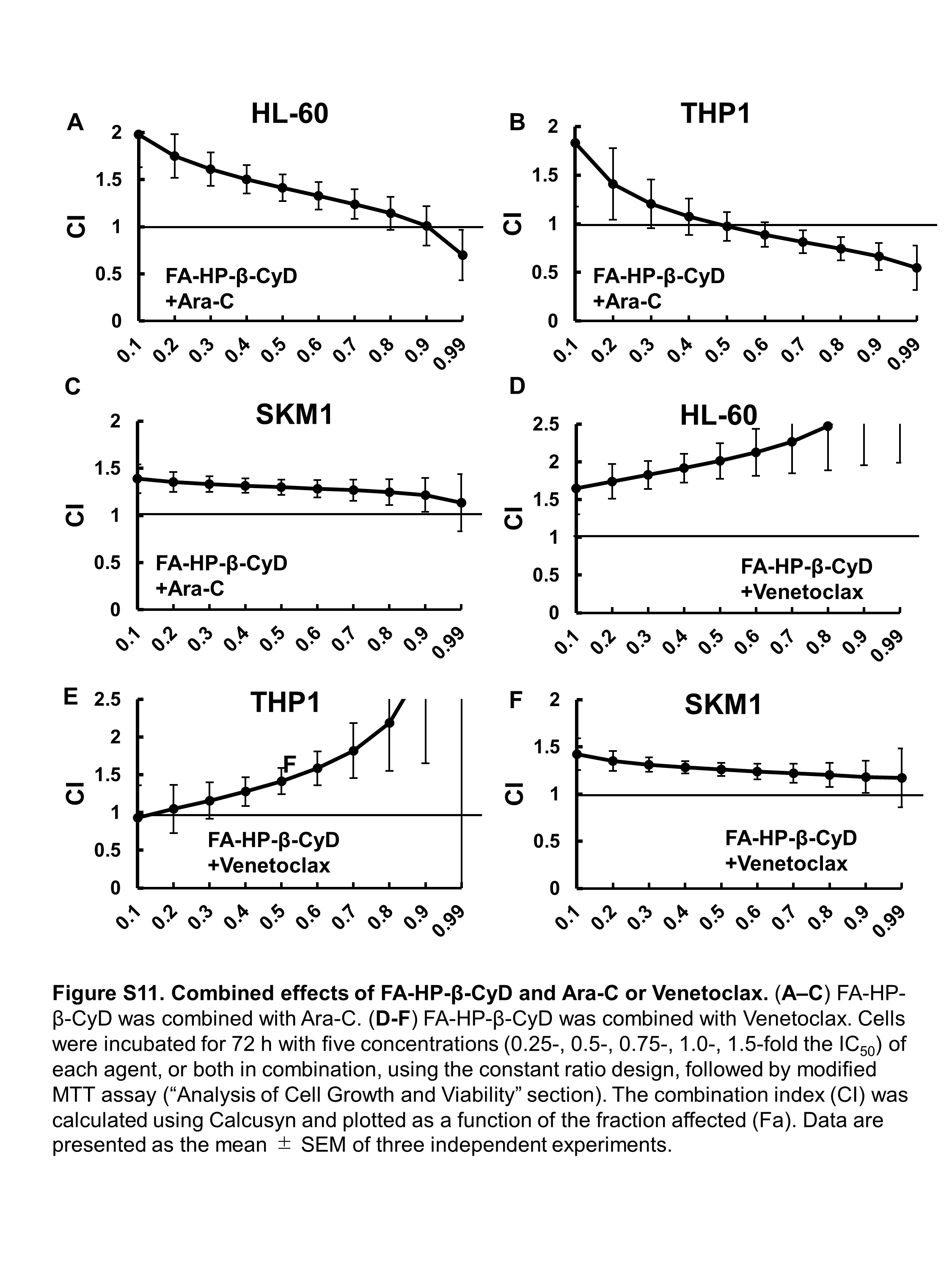

Supplement: Supplementary file 1 [file ijms-24-16720-s001.zip › Figure S11 proof.tif]

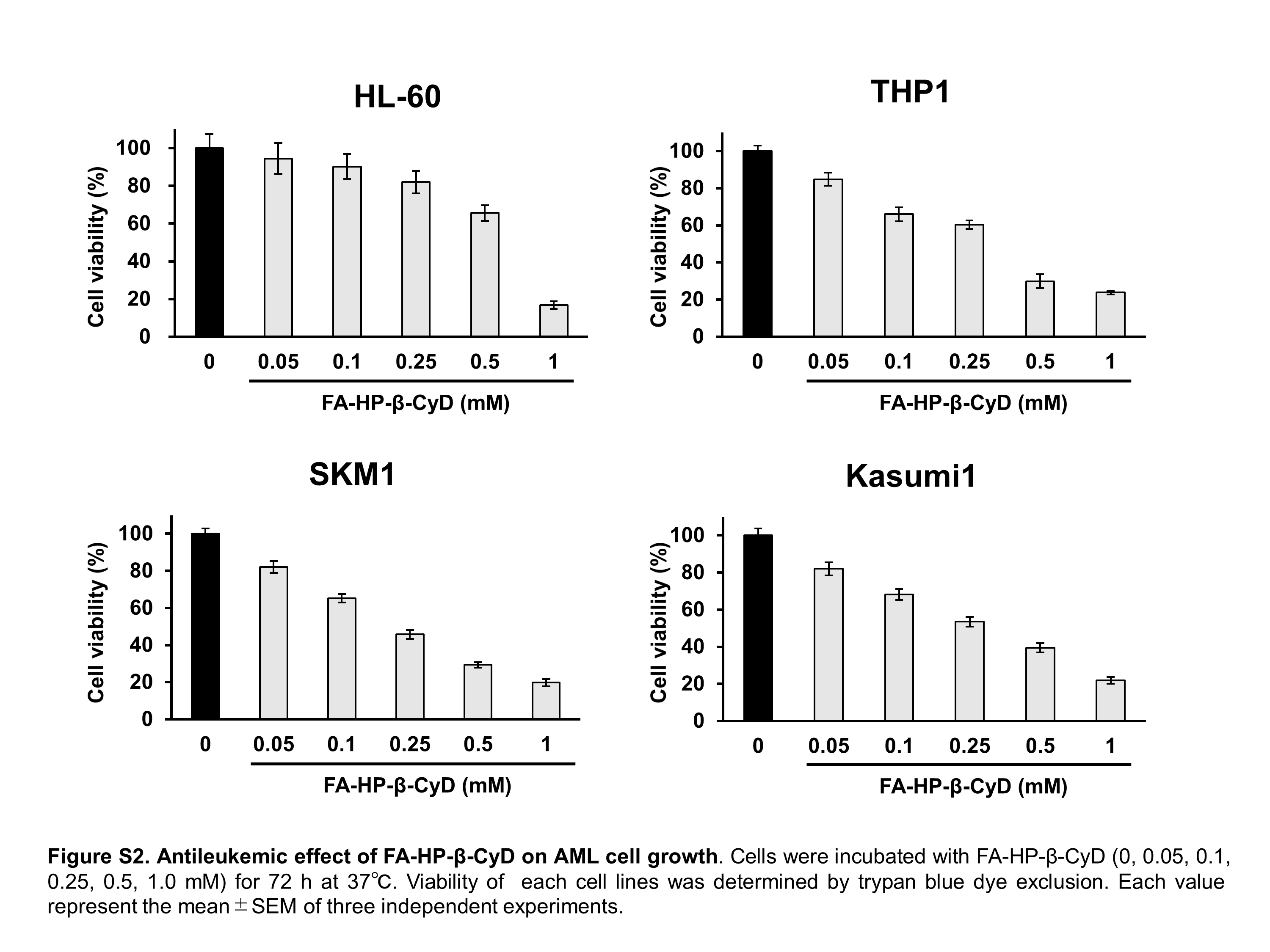

Supplement: Supplementary file 1 [file ijms-24-16720-s001.zip › Figure S2 proof.tif]

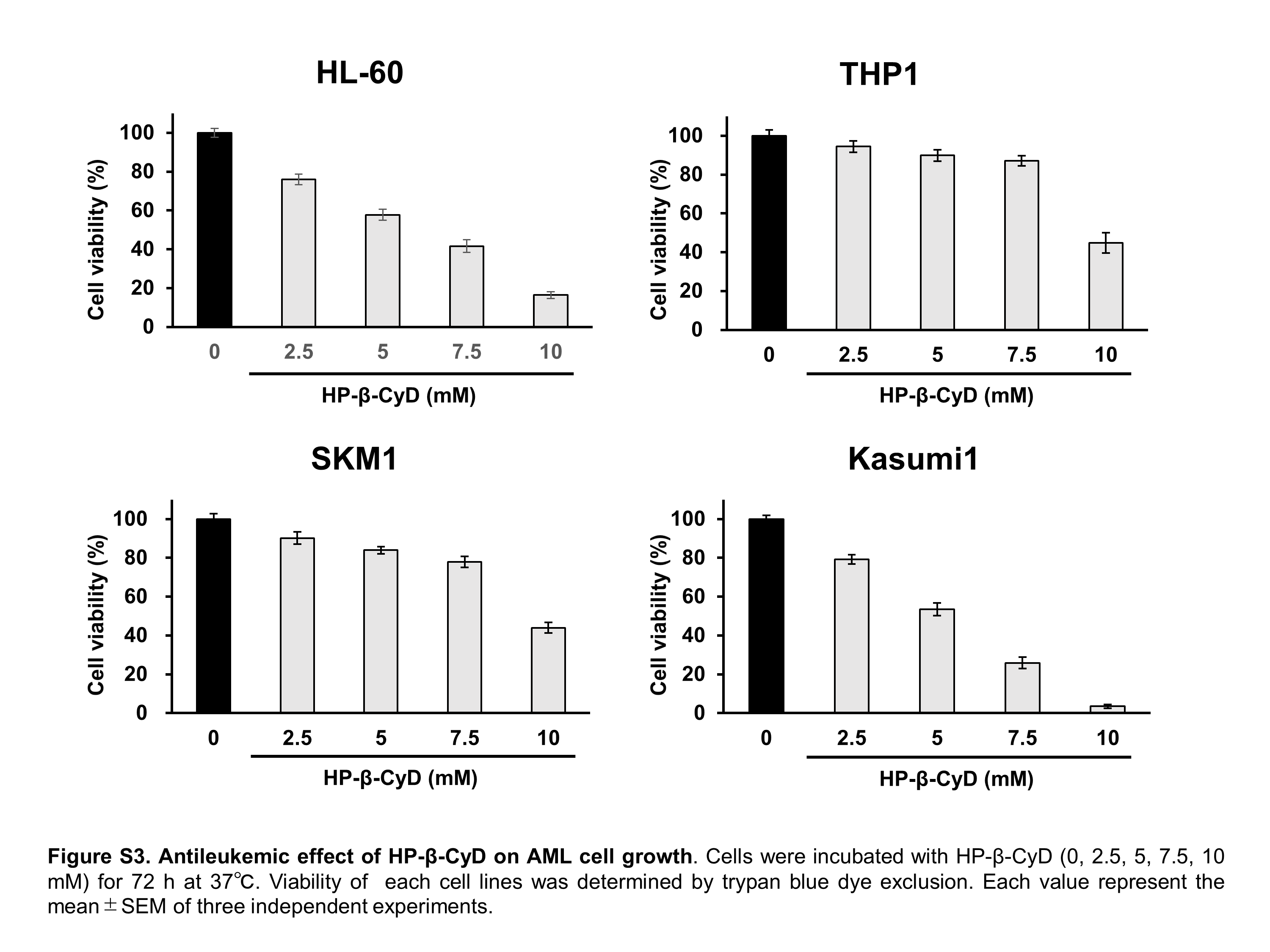

Supplement: Supplementary file 1 [file ijms-24-16720-s001.zip › Figure S3 proof.tif]

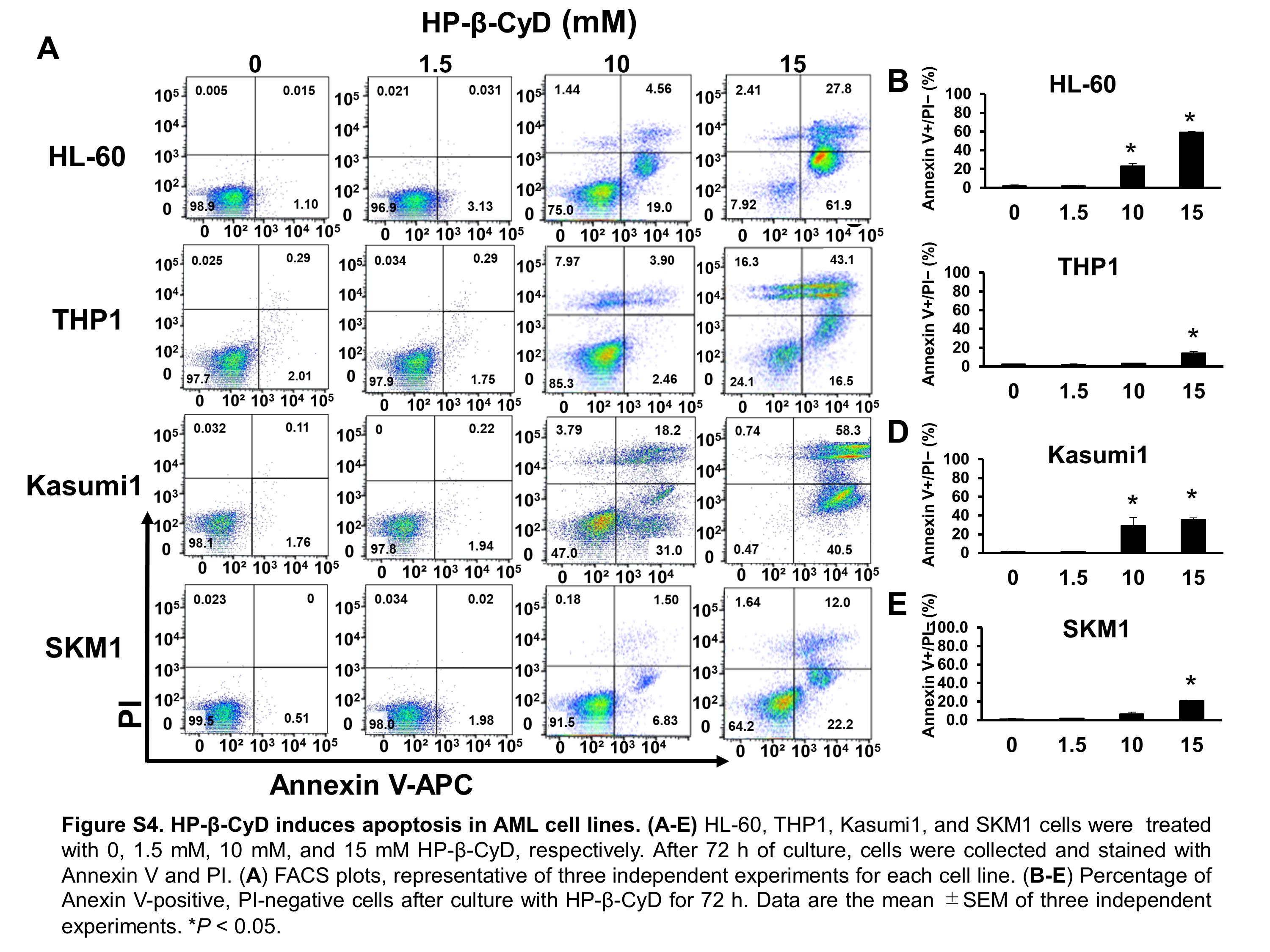

Supplement: Supplementary file 1 [file ijms-24-16720-s001.zip › Figure S4 proof.tif]

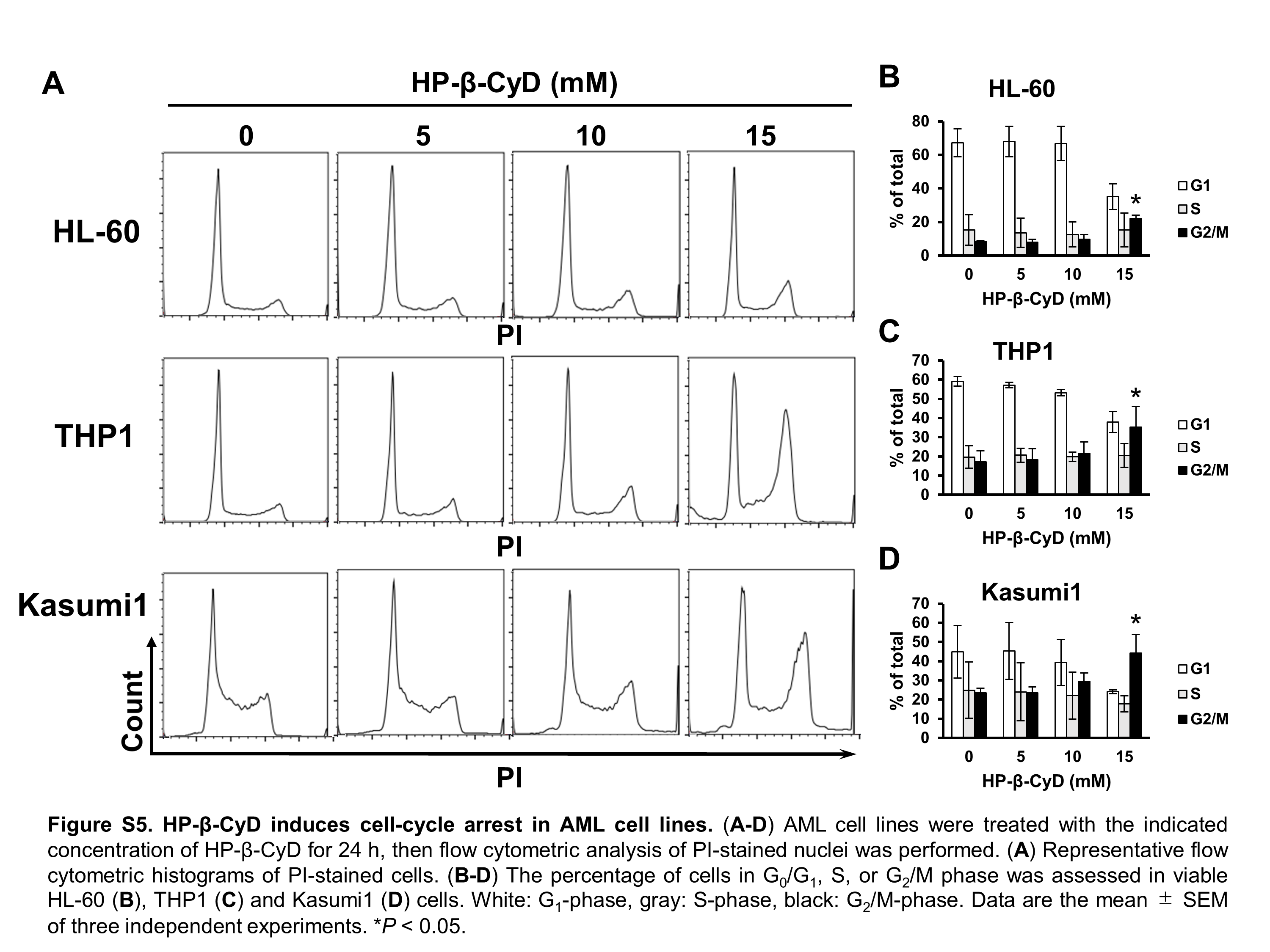

Supplement: Supplementary file 1 [file ijms-24-16720-s001.zip › Figure S5 proof.tif]

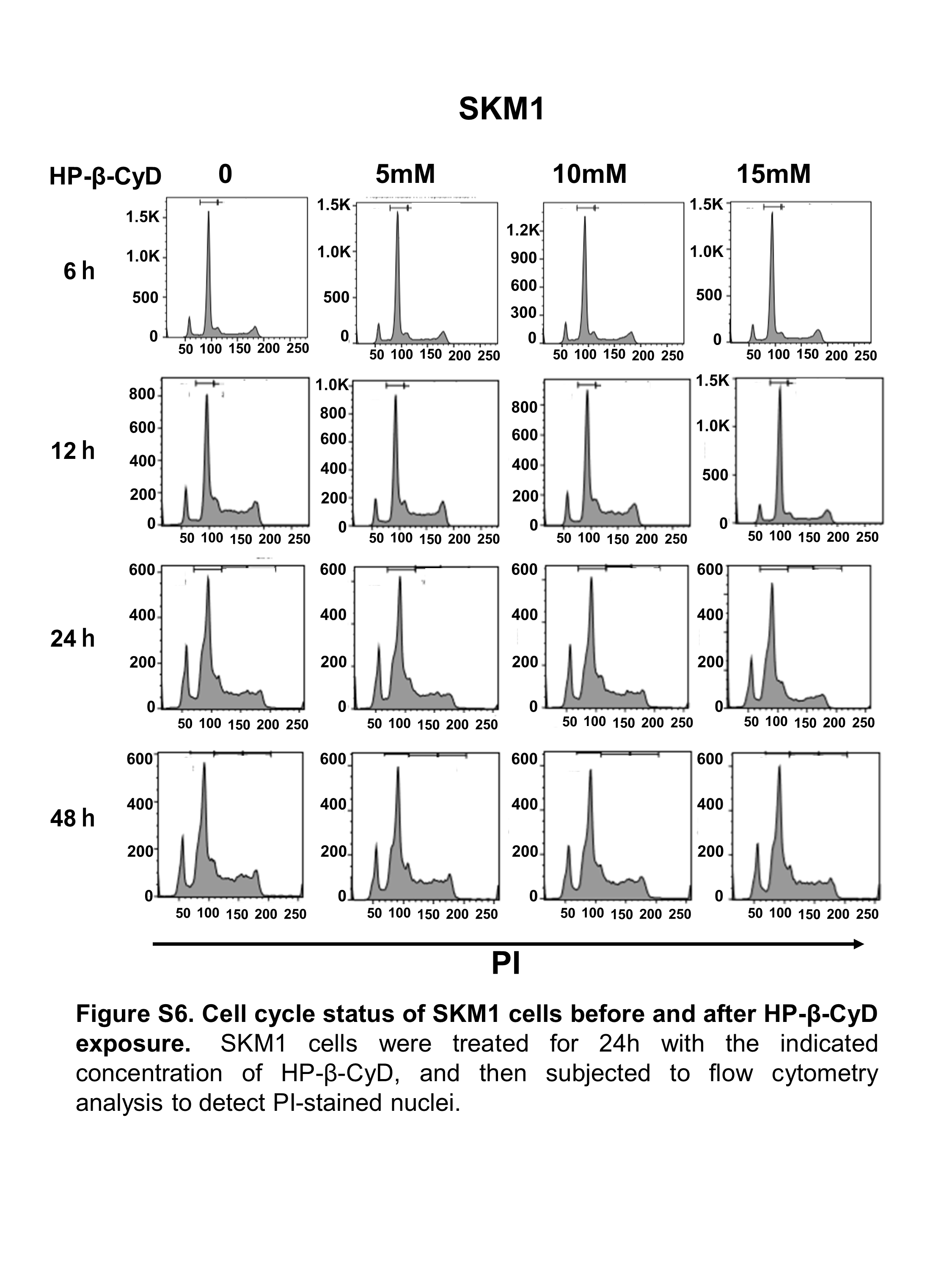

Supplement: Supplementary file 1 [file ijms-24-16720-s001.zip › Figure S6 proof.tif]

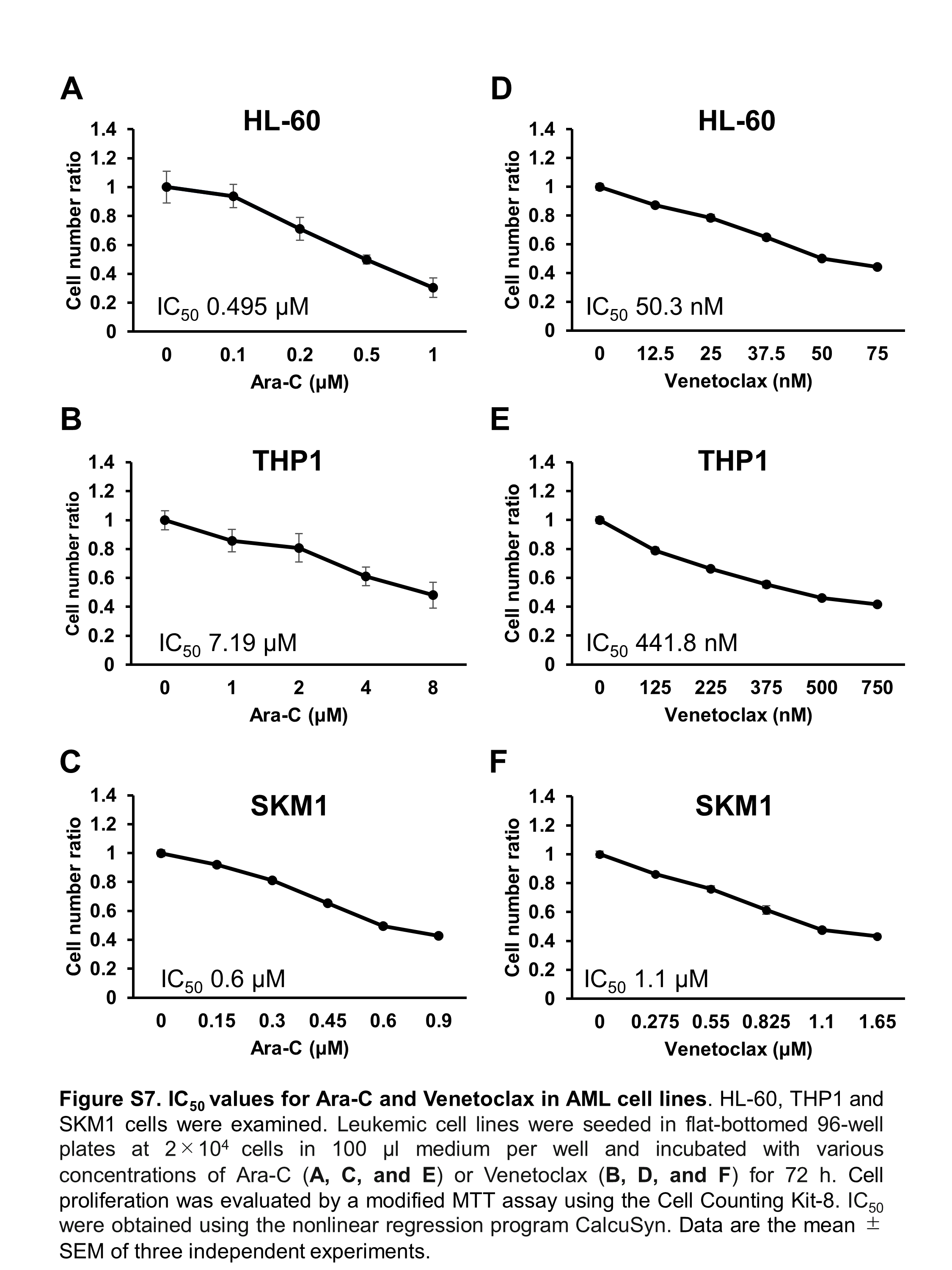

Supplement: Supplementary file 1 [file ijms-24-16720-s001.zip › Figure S7 proof.tif]

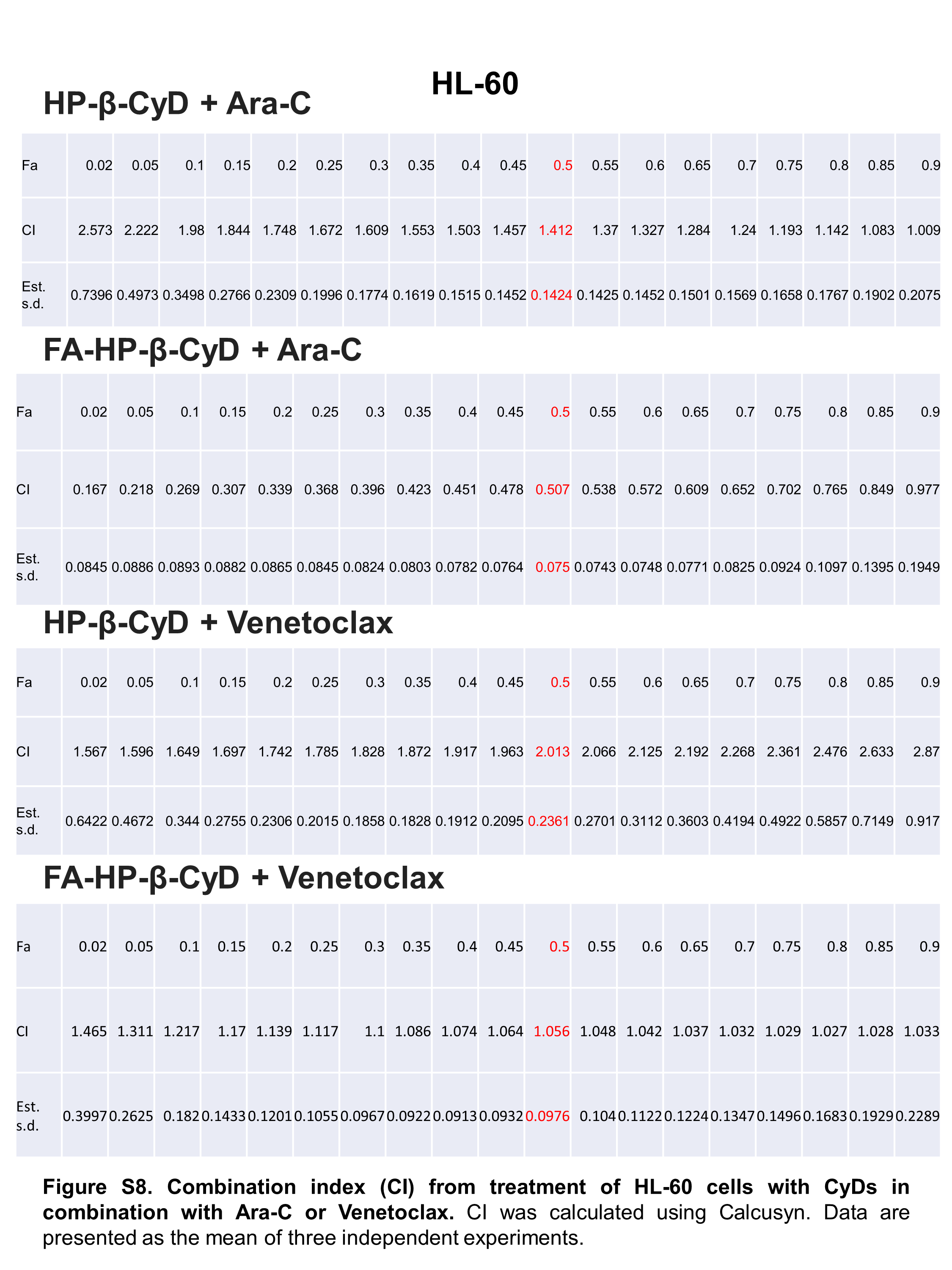

Supplement: Supplementary file 1 [file ijms-24-16720-s001.zip › Figure S8 proof.tif]

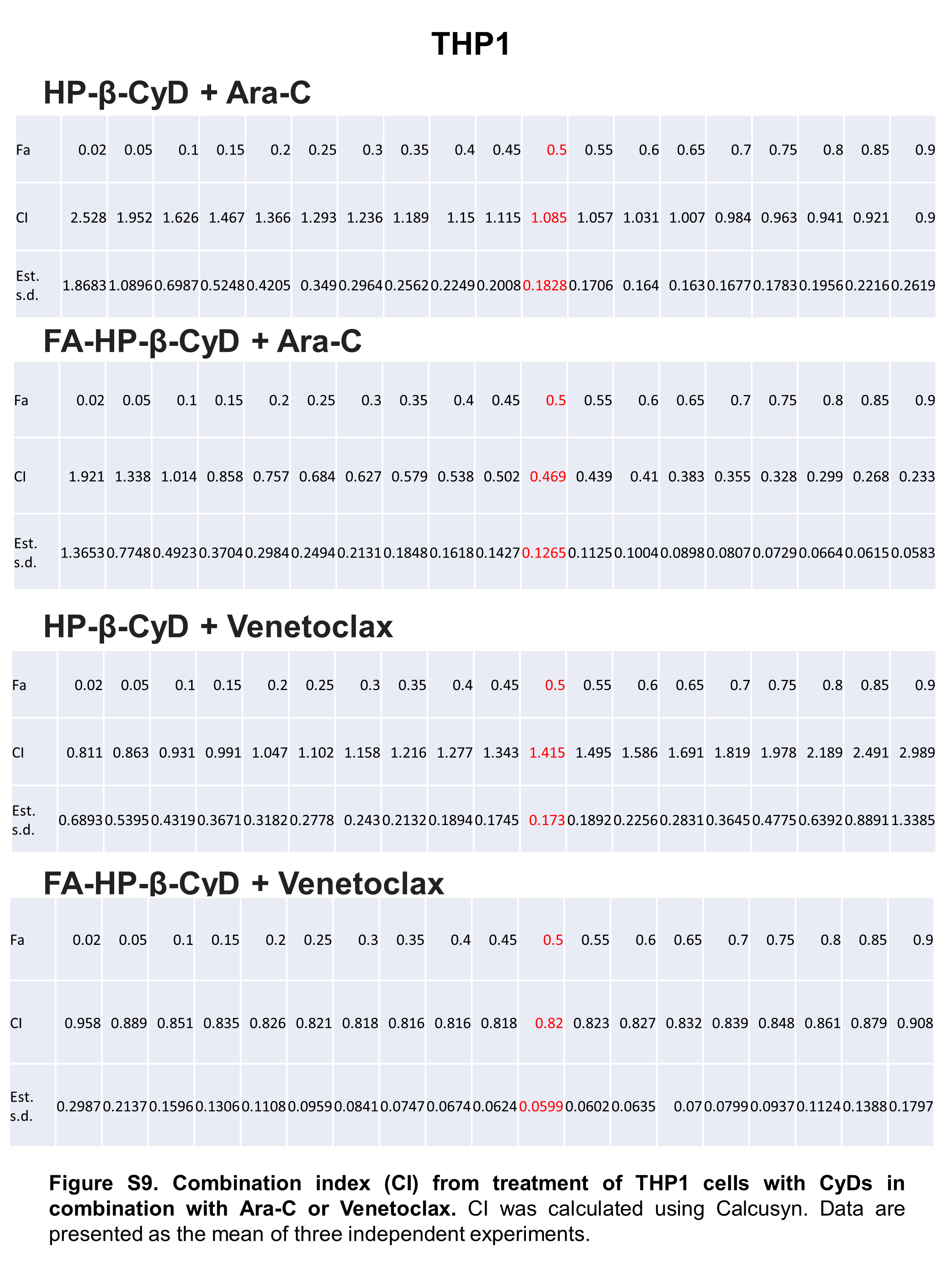

Supplement: Supplementary file 1 [file ijms-24-16720-s001.zip › Figure S9 proof.tif]
